# Method Validation and Assessment of Hazardous Substances and Quality Control Characteristics in Traditional Fruit Wines

**DOI:** 10.3390/foods11193047

**Published:** 2022-09-30

**Authors:** Chae-Wan Baek, Hyeon-Jun Chang, Jeung-Hee Lee

**Affiliations:** 1 Department of Food and Nutrition, Daegu University, Gyeonsan-si 38453, Gyeonsangbuk-do, Korea; 2 Fermented & Processed Food Science Division, Department of Agrofood Resources, National Institute of Agricultural Science, Wanju 55365, Korea

**Keywords:** fruit wine, acetaldehyde, cyanide, diacetyl, ethyl carbamate, method validation, sulfur dioxide

## Abstract

The presence of potentially hazardous substances in fruit wines poses a threat for human health. However, the management standards and specifications of hazardous substances contained within various types of fruit wines are currently insufficient. The aim of this study was to analyze hazardous substances (cyanide, acetaldehyde, and ethyl carbamate) and quality control characteristics (pH, titratable acidity, sulfur dioxide, and diacetyl) in seven different types of fruit wines. The pH levels and titratable acidity varied between fruit wine types. In all fruit wines, sulfur dioxide (SO_2_) was within acceptable ranges as per the Korean standard. Acetaldehyde content also varied between fruit wine types as well as based on the analytical method (titration or enzymatic analysis) employed. Cyanide was in the range of 0.02–0.35 mg/L. Diacetyl contents were in the range of 0.66–2.95 mg/L (*p* > 0.05). The contents of ethyl carbamate varied considerably, within the range of 5.22–259.69 μg/kg (*p* < 0.05). The analytical methods of diacetyl and ethyl carbamate were validated for specificity, linearity, sensitivity, accuracy, and precision. Therefore, the content of hazardous substances and quality control characteristics should be closely monitored and controlled to improve safety and quality of the traditional fruit wines.

## 1. Introduction

The global alcoholic beverage market is expected to grow by 19% from 2018 to 2024, and the wine market is to grow by 21% from 2018 to 2023 [1]. Fruit wine refers to wine fermented by adding fruits or sugars to fruits or wine that is filtered and conditioned by mixing fruit juice, carbon dioxide, and alcoholic beverages [2]. To reduce the waste of low-grade fruits and generate additional profit, a wide variety of fruits of different origins, cultivar, color, taste, and nutritional value are used for the production of wines worldwide [3]. Grape wine is the most popular among the fruit wines throughout the globe.

Apple, peach, and plum, the major fruits of Korea, are mainly consumed as raw fruits, meaning their utilization rate through processing is low. Recently, however, the consumer purchase rate has increased as fruit wine production has begun in earnest with the financial support of local governments and as online sales are now allowed. Apples are the most economically important and widely consumed fruit worldwide. In Korea, the export value of apple wine increased by 95.9% in 2020 compared to 2019, ranking it first among Korean traditional alcoholic beverages [1,3]. Peach ranks fourth in the world in terms of production volume after grape, apple, and pear. However, due to easy spoilage resulting from the loss of hardness during the aging process, it is mainly processed as canned food. As of recently, peach wine has been increasingly produced as a local specialty fruit wine [4]. On the other hand, maesil, a local specialty fruit, is processed into maesil wine, pickled salt, and pickled sugar due to its poor storage properties. Black raspberry (bokbunja) is also processed into a beverage, wine, and juice due to its small size and easy spoilage [5,6,7]. Although the production of maesil and black raspberry is comparably small, their popularity and recognition in Korea has led to their derivative fruit wines accounting for 67.0% and 55.5% of total processing for the respective fruits. That is, both fruits are mostly processed into wine, with market demand increasing steadily [7].

Alcoholic beverages (e.g., fruit wine, beer, and spirit drinks) are alcoholic liquors manufactured and processed by fermenting and distilling starch ingredients and contain an alcoholic strength of more than 1% [2]. Spirit drinks (e.g., rum, whisky, brandy, and vodka) have a minimum alcoholic strength by volume of 15% and are made via distillation [8]. Excessive alcohol intake is a major contributor to the burden of non-communicable diseases, such as cancer, cardiovascular disease, and liver disease [9]. The World Health Organization (WHO) International Agency for Research on Cancer (IARC) classified alcoholic beverages and the ethanol in alcoholic beverages as “carcinogenic to humans” (Group 1) [10]. Accordingly, the importance of controlling the quality characteristics and residual amounts of hazardous compounds in such beverages is paramount, with the WHO calling on governments to establish policies for the consumption of alcoholic beverages [11].

Hazardous compounds that may be contained in alcoholic beverages include acetaldehyde, cyanide, and ethyl carbamate. Acetaldehyde is mainly produced during alcohol fermentation by yeast, and when the activity of aldehyde dehydrogenase is suppressed, this compound can accumulate in the body, causing hepatitis, cirrhosis, and cancer [12]. Accordingly, the IARC has classified acetaldehyde associated with the consumption of alcoholic beverages into Group 1, ‘carcinogenic to human’ [10]. The European Union (EU) regulates the content of aldehydes (acetaldehyde) in distilled spirits at 0.5 mg/L or less [8]. The Korean Ministry of Food and Drug Safety (MFDS) imposes standards of acetaldehyde contents for distilled spirits and distilled alcoholic beverages at 10 mg/L and 700 mg/L, respectively [2]. However, there is no separate standard for limiting acetaldehyde content in fruit wines.

Cyanide (hydrogen cyanide) is produced from cyanogenic glycosides contained in cassava, apple, as well as apricot fruits, and can cause acute poisoning [13]. Further, it is linked to the causes of several chronic diseases, potentially leading to death in severe cases if consumed without proper processing [13]. Accordingly, the EU regulates hydrogen cyanide (HCN) content of fruit spirits and fruit marc spirits in spirit drinks at 70 mg/L or less [8]. China also regulates HCN content at 8 mg/L or less for distilled alcoholic beverages and 5 mg/L or less for distilled spirits [14]. Yet, Korea has not yet established a separate management standard for cyanide.

Ethyl carbamate is produced at low levels in alcoholic beverages as several precursors react with ethanol [15]. Hydrocyanide acid, as a precursor of ethyl carbamate, forms ethyl carbamate in fermented beverages (grape wine) or fruit wines made from stone fruits. On the other hand, citrulline produced from the catabolism of arginine also serves as a precursor of ethyl carbamate formation in grape wine and fruit brandies [16]. Since this substance can cause cancer by covalently binding to molecules such as DNA, RNA, and proteins, IARC has classified ethyl carbamate in group 2A, probably carcinogenic to humans’ [10]. Canada, the USA, and the Czech Republic regulate the ethyl carbamate concentration of wine at 30, 15, and 30 μg/L, respectively [15]. The EU recommended the prevention and reduction of ethyl carbamate contamination in stone fruit spirits and stone fruit marc spirits; however, the maximum levels for ethyl carbamate were not yet established [17]. Still, Korea has not set standards for ethyl carbamate content in alcoholic beverages and is yet to propose a test method and reduction plan for ethyl carbamate in respective alcoholic beverages [18].

Most of the research on fruit wines manufactured and distributed in Korea evaluates general quality characteristics (Brix, pH, color, ethanol, methanol, organic acid, and reducing sugar) or functional characteristics based on the types and contents of antioxidant and volatile compounds, mostly focusing on black raspberry, apple wine, red wine, and white wine [6,19,20,21]. On the other hand, there have been a limited number of studies comparing the contents of residual harmful substances between various traditional fruit wines manufactured in local communities.

In the present study, the residual amounts of harmful substances, such as acetaldehyde, cyanide, and ethyl carbamate, were determined in maesil wine and black raspberry wine as well as peach wine, apple wine, and plum wine, all of which are increasingly consumed as traditional Korean fruit wines. The values were then compared to those of imported wine. Quality control index values of Korean fruit wines, such as pH, total acidity, and contents of sulfur dioxide (SO_2_) and diacetyl, were analyzed and presented for quality improvement. In addition, method validation was carried out to evaluate the utility of analytical approaches employed for the characterization of fruit wines.

## 2. Materials and Methods

### 2.1. Samples, Chemicals, and Reagents

A total of five traditional fruit Korean wine types produced from maesil (*Prunus mume*), black raspberry (*Rubus coreanus* Miquel), peach (*Prunus persica*), apple (*Malus domestica*), and plum (*Prunus salicina*), as well as two types of imported red and white grape wines were purchased from local markets in Korea. Maesil wine is made from *Prunus mume*, which is commonly known as Japanese apricot or maesil in Korea. Black raspberry wine is made from *Rubus coreanus* Miquel, commonly known as Bokbunja in Korea. Red and white grape wine is made from *Vitis* *vinifera*. Ethyl carbamate (98% purity), butyl carbamate (98% purity), isonicotinic acid, potassium dihydrogen phosphate, sodium bisulfate (NaHSO_3_), thiosulfate solution (Na_2_S_2_O_3_), iodine solution (I_2_), tartaric acid, acetone, 1,2-diaminobenzene, diacetyl (2,3-butanedione, 99% purity), and 2,3-hexanedione (96% purity) were purchased from Sigma-Aldrich Co. Ltd. (St. Louis, MO, USA). Disodium hydrogen phosphate, chloramin T, and starch were purchased from Daejung Chemicals & Metals Co., Ltd. (Siheung, Korea). 3-methyl-1-phenyl-5-pyrazolone (pyrazolone), acetic acid, and phenolphthalein were obtained from Samchun Pure Chemicals Co., Ltd. (Pyeongtaek, Korea). Other solvents and reagents used were of analytical grade.

### 2.2. Analysis of pH Value and Titratable Acidity

The pH level was measured using a pH meter (Seven Compact S220, Mettler Toledo, Columbus, OH, USA). Titratable acidity was determined via a previously described titration method (AOAC method 947.05) [22]. Boiled and chilled distilled water (DW, 200 mL) was placed into a beaker and titrated to pH 8.2 by adding 0.1 N NaOH solution. The wine (5 mL) was then added and titrated again to pH 8.2, and the volume of 0.1 N NaOH solution (mL) used was recorded. Titratable acidity was calculated using Equation (1) and represented as tartaric acid (g/L).

(1) $$\mathrm{Titratable}\;\mathrm{acidity}\;\left(g/L\right)=\frac{V\;\times N\;\times f\;\times M}{\mathrm{Wine}\;\left(\mathrm{mL}\right)}\times 1000$$

where V = the volume of 0.1 N NaOH used for titration (mL); N = the normality of NaOH solution; f = the factor of 0.1 N NaOH; M = the milliequivalent factor of tartaric acid (0.075).

### 2.3. Analysis of Cyanide

The content of cyanide in fruit wines was analyzed based on Chinese National Food Safety Standards (GB 5009.36-2016) [14]. Wine (1mL) and 2 mL of NaOH solution (2 g/L) were added to a beaker and left for 10 min. The solution was concentrated to 1 mL at 120 °C, and the beaker was washed with NaOH solution (4 mL). After transferring the solution into a test tube, NaOH solution (1 mL of 10 g/L) and a few drops of 0.5% phenolphthalein solution were added and further adjusted by acetic acid solution (0.04%) until the red color started to fade. Two mL of phosphate buffer solution (0.5 M, pH 7.0) was then added and incubated at 37 °C for 10 min, whereafter 0.2 mL of chloramine T solution (1%) was added and reacted for 5 min. Two mL of isonicotinic acid-pyrazolone solution was then added [23]. Finally, samples were filled up to 10 mL by DW and incubated at 37 °C for 40 min. Absorbance was obtained at 638 nm using a microplate reader (Thermo Fisher Scientific Inc., Waltham, MA, USA). A calibration curve (potassium cyanide concentration of 0–0.3 mg/L) was used for quantification. Each wine sample was analyzed in triplicate, and the cyanide content was expressed as mg/L in wine.

### 2.4. Analysis of Total Sulfur Dioxide and Free Sulfur Dioxide

The contents of total sulfur dioxide (TSO_2_) and free sulfur dioxide (FSO_2_) in fruit wines were analyzed using total sulfur dioxide assay kits (K-TSULPH) and total and free sulfur dioxide assay kits (K-SULPH), respectively (Megazyme, Bray, Ireland), according to the manufacturer’s recommendations [24,25].

For TSO_2_ analysis, DW (1 mL), total sulfur dioxide reagent 1 (1 mL), and wine (0.05 mL) were mixed and reacted for 3 min. After that, the absorbance of solution (A_1_) was obtained at 405 nm using a microplate spectrophotometer (Thermo Fisher Scientific Inc., Waltham, MA, USA). Then, total sulfur dioxide reagent 2 (0.5 mL) was added, and the absorbance of the solution (A_2_) was obtained after 3 min. For blank analysis, the DW (0.05 mL) was used instead of wine. TSO_2_ standard solutions (0, 40, 80, 160, 320, and 400 mg/L) were prepared, and the above reaction was followed. After obtaining the absorbance of each standard, $△A_{{\mathrm{TSO}}_{2}\;\mathrm{of}\;\mathrm{std}}$ was calculated by subtracting the absorbance of the standard (0 mg/L) from the absorbance of each standard. The contents of TSO_2_ in fruit wines were determined using Equation (2):

(2) $$TSO_{2}\;\left(\mathrm{mg}/L\right)=\;\mathrm{mean}\;M\;\times F\;\times △A_{{\mathrm{TSO}}_{2}\;\mathrm{of}\;\mathrm{wine}}\;$$

where mean M: average value of M (each standard TSO_2_ concentrations divided by $△A_{T{\mathrm{SO}}_{2}\;\mathrm{of}\;\mathrm{std}}$); F: dilution factor; $△A_{T{\mathrm{SO}}_{2}\;\mathrm{of}\;\mathrm{wine}}$: (Blank A_2_ − Blank A_1_) − (Wine A_2_ − Wine A_1_).

For FSO_2_ analysis, DW 1 mL, free sulfur dioxide reagent 1 (1 mL), and wine (0.05 mL) were mixed. After 3 min, the absorbance of the solution (A_1_) was obtained at 575 nm. Free sulfur dioxide reagent 2 (0.5 mL) was added, after then the absorbances (A_2_ and A_3)_ of solution were obtained at 3 min-intervals, respectively. For calculation of FSO_2_ content, FSO_2_ standard solution (0, 15, 30, 60, 120, and 150 mg/L) was prepared, and absorbance (A_2_) of each FSO_2_ standard was obtained. Next, $△A_{F{\mathrm{SO}}_{2}\;\mathrm{of}\;\mathrm{std}}$ was calculated by subtracting the absorbance of standard (0 mg/L) from the absorbance of each standard, respectively. The content of FSO_2_ was calculated using Equation (3):

(3) $$FSO_{2}\;\left(\mathrm{mg}/L\right)=\mathrm{mean}\;M\;\times F\;\times △A_{{\mathrm{FSO}}_{2}\;\mathrm{of}\;\mathrm{wine}}\;$$

where mean M: average value of M (each standard FSO_2_ concentrations divided by $△A_{F{\mathrm{SO}}_{2}\;\mathrm{of}\;\mathrm{std}}$; F: dilution factor; $△A_{F{\mathrm{SO}}_{2}\;\mathrm{of}\;\mathrm{wine}}$: {(Blank A_2_ − Blank A_1_) − (Blank A_3_ − Blank A_2_)} − {(Wine A_2_ − Wine A_1_) − (Wine A_3_ − Wine A_2_)}. TSO_2_ and FSO_2_ content was represented as mg/L in wine.

### 2.5. Analysis of Acetaldehyde

Acetaldehyde contents in fruit wines were analyzed using the titration method and enzymatic assay. For titration method (MFDS 8.6.7.2.1 aldehyde-2022), DW (45 mL), wine (5 mL), and 0.01 N NaHSO_3_ (corresponding to 10 mL of 0.005 M I_2_) were mixed in a beaker and then left for 30 min [2]. Then, 0.005 M I_2_ (10 mL) and 1% soluble starch solution (1 mL) were mixed and titrated with 0.01M Na_2_S_2_O_3_ (A mL) until the blue-purple color started to fade. For blank analysis, DW (5 mL) was used instead of wine and titrated (B mL). The acetaldehyde contents were calculated via the following Equation (4):

(4) $$\mathrm{Acetaldehyde}\;\left(\mathrm{mg}/L\right)=\frac{\left(A-B\right)\times F\;\times 0.22}{\mathrm{Wine}\;\left(\mathrm{mL}\right)}\times 1000$$

where A: 0.01 N Na_2_S_2_O_3_ titration volume for wine (mL); B: 0.01 N Na_2_S_2_O_3_ titration volume for blank (mL); F: 0.01 Na_2_S_2_O_3_ factor.

For the enzymatic assay, acetaldehyde content was analyzed using acetaldehyde assay kits (K-ACHYD, Megazyme, Bray, Ireland) according to the manufacturer’s protocols [26]. Briefly, DW (2 mL), wine (0.1 mL), buffer solution plus sodium azide (0.2 mL, 0.02% w/v), and NAD^+^ solution (0.2 mL) were mixed and reacted for 2 min. The absorbance of the solution (A_1_) was obtained at 340 nm using a microplate spectrophotometer (Thermo Fisher Scientific, Multiskan, GO, USA). Then, aldehyde dehydrogenase solution (0.05 mL) was added, and the absorbance of the solution (A_2_) was obtained after 4 min. Acetaldehyde standard solution (0.1–200 mg/L) was prepared, and the calibration curve was obtained and used for the quantification of acetaldehyde. The acetaldehyde content was expressed as mg/L of wine. Each wine sample was analyzed in triplicate.

### 2.6. Analysis of Ethyl Carbamate

Wine (5 g) was mixed with 1 mL of butyl carbamate solution (400 ng/mL, internal standard), and DW was added until a weight of 40 g was obtained. This diluted wine mixture was loaded onto the diatomite solid phase extraction column (50 mL, Chem Elut^TM^, Agilent Technologies Inc., Santa Clara, CA, USA) and left for adsorption (4 min). The column was extracted twice with 80 mL of dichloromethane, and the eluent was concentrated with a rotary vacuum evaporator (N-1110, Sunil Eyela, Seongnam, Korea), dried into 1 mL under a stream of N_2_, and then subjected to Gas Chromatography-Mass Spectrometry (GC-MS) analysis.

The GC/MS-QP2100 Plus (Shimadzu Corp., Kyoto, Japan) equipped with DB-WAX (30 m × 0.25 mm I.d, 0.25 µm film thickness, Agilent Technologies Inc., Santa Clara, CA, USA) was used for analysis of ethyl carbamate according to the method of Qin et al. [23], with some modifications. The carrier gas was helium at a flow rate of 0.9 mL/min. An aliquot of 1 μL of wine sample was injected in splitless mode, and injector temperature was set at 180 °C. The oven temperature was programmed as follows: 40 °C (0.75 min) → 60 °C (10 °C/min) → 150 °C (3 °C/min, 150 °C for 3 min) → 220 °C (4.25 min). The temperatures of transfer line and ion source were set at 230 °C. The MS was operated in selected ion monitoring (SIM) mode with electron impact ionization (70 eV), using the ions of 62, 74, and 89 m/z for the qualification of ethyl carbamate. For quantification, 62 m/z was used.

Each standard stock solution of ethyl carbamate and butyl carbamate was prepared at a concentration of 1 mg/mL in acetone. Standard solutions of ethyl carbamate (0.39–1600 ng/mL in dichloromethane) with butyl carbamate (400 ng/mL) were prepared to obtain a calibration curve. The ethyl carbamate content was calculated and expressed as μg/Kg of wine. Each wine sample was analyzed in triplicate.

### 2.7. Analysis of Diacetyl

Diacetyl content in fruit wines was analyzed as per the method described by the International Organization of Vine and Wine (OIV-MA-AS315-20: R2010) [27]. The wine (10 mL) was placed into a 25 mL vial, and the pH was adjusted to 8.0 with 0.05 M NaOH solution. Fifty μL of internal standard solution (2,3-hexanedione, 0.934 μg/mL in 50% ethanol) was added and derivatized with 5 mg of 1.2-diaminobenezene (OPDA) at 60 °C for 3 h in a shaking water bath (Daihan Labtech Co., Namyangju, Korea). The vial was cooled, and the diacetyl content was analyzed with High-Performance Liquid Chromatography-Ultraviolet (HPLC-UV, Shimadzu Corp., Kyoto, Japan) equipped with ZORBAX Eclipse XDB-C18 column (250 m × 4.6 mm, 5 μm particle, Agilent, Santa Clara, CA, USA). The injection volume was 20 μL. The flow rate of the mobile phase was set at 0.6 mL/min, and the UV wavelength was set at 313 nm. Gradient elution was carried out using water-acetic acid (100:0.05, v/v) as solvent A and methanol as solvent B with the following gradient: 0 to 8 min (80% A), 8 to 26 min (50% A), 26 to 30 min (25% A), 30 to 40 min (0% A), 40 to 45 min (100% A), 45 to 50 min (80% A). The calibration curve was obtained using diacetyl standard solution (0.098–19.620 μg/mL prepared by 10% ethanol) with internal standard. The diacetyl content was calculated and expressed as mg/L of wine. Each wine sample was analyzed in triplicate.

### 2.8. Method Validation

Analytical methods were validated based on specificity, linearity, accuracy, precision, and sensitivity in accordance with the International Council for Harmonization (ICH) guideline [28].

#### 2.8.1. Specificity and Linearity

The specificity of analytical methods was validated with internal and external standards, in which the peaks of diacetyl and ethyl carbamate were identified with retention time in chromatograms obtained by HPLC-UV and GC-MS-SIM, respectively. Linear regression analysis was used to evaluate the linearity of calibration curves, and the related linearities (coefficients of determination, R^2^) were obtained.

#### 2.8.2. Accuracy and Precision

The accuracy of methods was determined by spike and recovery testing, where the known concentrations of analytes (acetaldehyde, diacetyl, and ethyl carbamate) at different concentrations were spiked into the selected wines (maesil wine, apple wine, and white grape wine). Their contents were measured in triplicate. The percentage recovery was calculated using the following Equation (5):

(5) $$\mathrm{Recovery}\;\left(\%\right)=\frac{X1-X2}{S}\times 100$$

where X1 = analyte concentration in the spiked wine; X2 = analyte concentration in wine; S = spiked analyte concentration.

The precision of analytical method was determined by repeatability and expressed as the relative standard deviation (RSD%).

#### 2.8.3. Sensitivity

The sensitivity of methods was determined based on limit of detection (LOD) and limit of quantification (LOQ). The LOD and LOQ for diacetyl using HPLC-UV were determined based on the ICH guideline [28]. The peak areas on six blank samples from HPLC chromatograms were averaged and multiplied by 3.3 and 10 to obtain the concentrations of A and B. The calibration curve was prepared with five concentrations, including the concentration of A and B, by plotting concentration against the peak area. The LOD (3.3 × SD/S) and LOQ (10 × SD/S) were calculated, in which SD = the standard deviation; S = the slope of the calibration curve. The LOD and LOQ of ethyl carbamate determined via GC-MS-SIM were obtained from the standard deviations of y-intercepts and slopes of the calibration curves. Calibration curves were constructed to verify linearity, and the standard deviations and slopes were calculated from the calibration curves [29].

### 2.9. Statistical Analysis

Analysis of variance (ANOVA) was performed using Statistical Analysis System 9.2 (SAS Institute, Inc., Cary, NC, USA). The statistical differences between the two analytical methods for acetaldehyde and between the contents of harmful substances from two plum wines were determined by Student’s *t*-test at *p* < 0.05. The statistical difference of means between more than three groups was determined using Duncan’s multiple range test at *p* < 0.05. The correlation between TSO_2_ and acetaldehyde contents in wines was determined via the Pearson correlation coefficient (r) using SPSS Version 25.0 (IBM Corp., Armonk, NY, USA). *p* < 0.05 and *p* < 0.01 were considered indicative of statistical significance.

## 3. Results and Discussion

### 3.1. pH Level and Titratable Acidity in Fruit Wines

The pH level was highest in peach wine (3.80–3.81), followed by apple, plum, red grape, white grape, maesil, and black raspberry wines (Table 1). The titratable acidity was highest in peach wine (6.74–8.09 g/L), followed by plum, black raspberry, white grape, red grape, maesil, and apple wines, with that of peach and plum wines being 1.17 to 1.70 times higher than those of other fruit wines (*p* < 0.05) (Table 1).

Maesil, black raspberry, peach, apple, and plum fruits contain organic acids, such as oxalic, malic, citric, and tartaric acid, with contents varying between fruit types [30,31,32,33,34]. The pH and acidity of fruit wines are affected by the type and concentration of organic acids produced by microorganisms during the fermentation process of fruit or ethyl alcohol [35]. pH of grape wine is a factor that affects the fermentation process, storability, and taste. At a pH of 3.6 or greater, contamination by various bacteria (e.g., *Pediococcus* and *Lactobacillus*) may occur [35,36]. On the other hand, if the pH is 3.2 or lower, the organoleptic quality may decrease due to bitterness. Thus, a pH of 3.2 to 3.5 is recommended for the production and storage of grape wine [35,37]. In the present study, the pH of imported red grape wine and white grape wine was 3.25 to 3.64, whereas the pH of traditional Korean fruit wines was in the range of 2.96 to 3.84. The pH levels of respective fruits were reported as follows: pH 2.51 to 2.76 for maesil [30], pH 3.43 to 3.52 for black raspberry [31], pH 3.41 to 4.17 for peach [32], pH 4.42 to 4.80 for apple [33], and pH 3.2 to 3.5 for plum [34]. In previous studies, the pH levels of fruit wines were reported as 2.91–3.11 for Japanese apricot wine (maesil wine) [20], 3.58–3.64 for Korean raspberry wine [31], 3.44–3.54 for peach wine [38], 4.22–4.26 for apple wine [39], 3.4–3.5 for plum wine [40], 3.47–3.57 for red wine [20], and 3.17–3.38 for white wine [20].

Acidity is not only an indicator for the evaluation of fruit wine quality characteristics or rancidity but also an important factor in determining taste [20]. If acidity is high, an abnormal fermentation process is predicted, and the wine may have a rough and strong sour taste. However, low acidity results in a fruit wine that is plain and easily spoiled during storage [40,41]. The recommended acidity of grape wine is from 6 to 6.5% [42]. Acidity in fruit wine is mainly contributed by the presence of organic acids; these are naturally produced by microorganisms or added to alcoholic beverages as preservatives, antioxidants, pH adjusters, acidulants, and flavoring agents [43,44,45]. In particular, tartaric acid is added to fruit wine lacking acidity due to the resistance to degradation and metabolization by wine microorganisms [44]. Malic acid is a major factor in determining the taste and quality of grape wine. However, high levels of malic acid can provide bitterness, and therefore, malolactic fermentation is necessary to reduce malic acid during the fermentation of grape wine [42]. In the present study, based on the ingredients listed by the fruit wine manufacturer, tartaric acid, malic acid, and citric acid had been added. Taken together, it seems that the pH and acidity of fruit wines are influenced by the organic acid content of the raw material itself, organic acids generated by microorganisms during the fermentation process, and by organic acids added directly or indirectly to the product to control the pH.

In previous studies, total acidity in peach wine, plum wine, maesil wine, and grape wines was reported at 8.0–9.6 g/L, 6.7–8.6 g/L, 4.8–6.9 g/L, and 4.6–6.3 g/L, respectively [20,38,40]. In Korea, the standard total acidity content of distilled spirits is 0.002 w/v% or lower, but separate pH and total acidity quality standards for fruit wines have not been established [2]. Therefore, to prevent contamination and deterioration during the storage and distribution of fruit wines as well as to enhance their sensory characteristics, it is necessary to introduce appropriate regulatory thresholds.

### 3.2. The Content of Cyanide in Fruit Wines

Cyanogenic glycosides (linamarin, lotaustalin, dhurrin, amygdalin, and prunasin) are HCN-producing phytotoxins formed by amino acids (valline, isoleucine, leucine, tyrosine, and phenylalanine) in several plant species, such as cassava, peach, apple, and maesil [13,46]. Cyanogenic glycosides are not toxic by themselves, but when plant tissue is disrupted, they are hydrolyzed and release hydrogen cyanide (HCN), which is toxic to humans and animals [13]. In the fruits of maesil, peach, apple, plum, and grape, cyanogenic compounds occur the most in seeds (trace–1091.02 mg/L), whereas trace amounts are contained in the skin (trace–2.65 mg/L) and flesh (trace–0.41 mg/L). Among fruits, maesil has the highest content [47]. Amygdalin, a cyanogenic glycoside of maesil is hydrolyzed into prunasin and mandelonitrile by β-glucodidase to yield glucose, benzaldehyde, and HCN [48].

During wine manufacture, entire fruits with flesh and seeds are normally used, with the cyanogenic glycosides transferred into wine during the steeping process. Further, the alcohol can lead to hydrolysis of cyanogenic glycoside into HCN. The content of cyanogenic glycosides (as amygdalin) in wines depends on the levels in respective fruits (i.e., maesil, apricot, and apple) as well as on several factors, including temperature, time of extraction, alcohol concentration, and the ratio of solid to liquid [49]. The cyanogenic glycosides in fruit wine were significantly lower than corresponding fruits. The cyanogenic glycoside content in apricot and cherry liqueurs was reported at 38.79 µg/mL and 16.08 µg/mL, respectively, during steeping process, consistently decreasing thereafter [50]. However, because the released glycosides are degraded into harmful HCN, studies should focus on raw material selection, fermentation, and maturation process for reducing cyanide in fruit wine.

In the present study, red grape wines (ave. 0.35 mg/L) exhibited considerably higher cyanide content than the other fruit wines (*p* < 0.05), whereas white wine exhibited the lowest cyanide content (0.02 mg/L) (Table 2). Among traditional fruit wines, cyanide content was highest in apple wine (0.13 mg/L), followed by black raspberry and plum wines (0.08 mg/L) as well as maesil and peach wines (0.06 mg/L), with no significant difference among these (*p* > 0.05). The cyanide content of red grape wine was 17.5 times higher than that of white grape wine. For white grape wine, the fermentation process is carried out after removing the skins and seeds of grapes, whereas in red grape wine, the fermentation process is carried out on the grape itself. Therefore, it is thought that red grape wine has a higher cyanogenic glycoside content released from grape seeds than white grape wine, and HCN formation is increased due to degradation [49].

### 3.3. The Content of Total and Free Sulfur Dioxide in Fruit Wines

Sulfur dioxide (SO_2_) is added during wine manufacture for the purpose of suppressing microorganisms, preventing oxidation, color stabilization, and control of enzymatic browning [50,51]. SO_2_ is safe for most people, but rare hypersensitivity reactions can cause bronchoconstriction, headache, and abdominal pain in certain individuals [52]. EU regulations limit the concentration of TSO_2_ to 150 mg/L in red grape wine and 200 mg/L in white grade wine [53]. Korea regulates the use of SO_2_ as a food additive for fruit wines at 350 mg/L or lower [54]. Most of the SO_2_ contained in wine is combined with carbonyl compounds (diacetyl, acetaldehyde, pyruvate), existing in the form of bound SO_2_, while free SO_2_ usually exists in the form of HSO_3_^−^, and total SO_2_ content is expressed as the sum of free and bound forms [20,50,55].

The contents of TSO_2_ and FSO_2_ in the fruit wines are presented in Table 3. The TSO_2_ range varied between maesil, peach, apple, plum, red grape, and white grape wines, whilst not detected in black raspberry wine. FSO_2_ content was greatest in apple, followed by peach, white grape, red grape, and plum wines, while not detected in both maesil and black raspberry wines. The ratio of FSO_2_ to TSO_2_ ranged from 0.17 to 7.95% in peach wine, 0 to 11.79% in apple wine, 0.53 to 3.60% in plum wine, 21.05 to 89.63% in red grape wine, and 2.07–11.69% in white grape wine. We confirmed a TSO_2_ of 350 mg/L or less in all fruit wines, which is within the Korean MFDS standard. Comparing analytical results and the wine ingredients provided by the manufacturer, TSO_2_ and FSO_2_ were not detected in fruit wines (three black raspberry wines and one apple wine) that had no labeling of SO_2_ addition. On the other hand, there were fruit wines (two maesil wines) labeled for SO_2_ addition, wherein none was detected. SO_2_ in fruit wines (except black raspberry wine) exists in both bound and free forms. Shin and Lee [20] reported that the TSO_2_ content of maesil wine was 4.48 to 23.10 mg/L, while FSO_2_ was not detected, leading to the conclusion that most SO_2_ existed in the bound form. A previous study reported that TSO_2_ levels of Korean fruit wines were in the range of 7.17 (raspberry wine) to 120.57 mg/L (kiwi wine), while the TSO_2_ content of grape wine was 94.86 mg/L [55].

TSO_2_ and FSO_2_ contents of white grape wine were 12.78 times and 2.33 times higher than those of red grape wine, respectively. Shin and Lee [20] found that the TSO_2_ content of red and white grape wine was 26.60 to 45.98 mg/L and 92.73 to 158.98 mg/L, respectively, while the FSO_2_ content was 4.68 to 8.27 mg/L and 11.85 to 36.93 mg/L, respectively. Cho et al. [55] also reported that the TSO_2_ content of red wine was significantly lower than that of white wine, similar to the results of our study. In the wine fermentation process, red grape wine has a longer contact time with grape skins than white grape wine. Thus, red grape wine has a much higher concentration of polyphenols than white grape wine because the spectrum of polyphenols extracted from grape skin is broader [55]. Therefore, it is considered that the TSO_2_ and FSO_2_ content in white wine is higher than that of red wine because a larger amount of SO_2_ is added to white grape wine for the antioxidant effect of wine and to control the browning reaction.

### 3.4. The Content of Acetaldehyde in Fruit Wines

Acetaldehyde is mainly produced during alcohol fermentation by yeast and is classified into Group 1, ‘carcinogenic to human’ [10]. Acetaldehyde plays a major role in the color development of wine and gives a pleasant fruity aroma as a flavor compound at low concentrations. At excessive concentration, a pungent irritating odor reduces the taste and aroma of alcoholic beverages but can be masked by adding SO_2_ [56,57]. Over 80% of the acetaldehyde in wine is bound to SO_2_, and acetaldehyde quantification can be affected by whether and how much SO_2_ was added [58].

The acetaldehyde content of fruit wines, as determined via the titration method, was highest in plum wine (19.04–74.68 mg/L), followed by maesil, black raspberry, red grape, peach, and white grape wines (Table 3). The acetaldehyde content of fruit wines determined via enzymatic assays was highest in peach wine (14.47–108.38 mg/L), followed by apple, white grape, plum, maesil, red grape, and black raspberry wines (Table 3). Comparing acetaldehyde quantification via the two analytical methods, the titration method determined higher contents in maesil wine, black raspberry, and plum wines, whereas the enzymatic assay detected higher contents in peach wine and apple wine, with no significant difference between methods (*p* > 0.05). On the other hand, red grape wine was found to have a significantly higher acetaldehyde content via the titration method and white grape wine via the enzymatic assay (*p* < 0.05). In a previous study, acetaldehyde in red wine was determined at 21.33 to 33.33 mg/L (by titration) and 20.95 to 25.23 mg/L (by enzymatic assay), whilst at 4.00 to 16.00 mg/L (by titration) and 37.53 to 68.64 mg/L (by enzymatic assay) in white wine, which is in agreement with the current results [20].

Acetaldehyde binds to SO_2_ and flavonoids (catechin, anthocyanin, and tannin) present in fruit wine to form polymers such as acetaldehyde-bound SO_2_ and acetaldehyde-bound flavonoids with ethylidene bridges, respectively [20,57,59]. During analysis via the titration method, wine acetaldehyde interacts with the sodium bisulfate solution added, and the sodium bisulfate-acetaldehyde formed at this step is titrated with sodium thiosulfate solution to calculate acetaldehyde content. Acetaldehyde bound to SO_2_ and flavonoids cannot form sodium bisulfate-acetaldehyde, so it can underestimate the amount actually present in wine [20]. A significant amount of TSO_2_ is contained in peach wine (39.94–302.75 mg/L), apple wine (144.95–148.38 mg/L), and white grape wine (79.88–121.33 mg/L), yet acetaldehyde content determined via the titration method was significantly lower than that determined via the enzymatic assay (*p* < 0.05). In addition, the Pearson correlation coefficient (r) between TSO_2_ and acetaldehyde contents in wines was r = − 0.127 (negative correlation) for the titration method and r = 0.931 (positive correlation) for enzymatic assay (data in Supplementary File). Therefore, the acetaldehyde quantification of fruit wine with high SO_2_ content is affected by the analysis method, with the enzymatic assay method considered more appropriate for this study.

### 3.5. The Content of Diacetyl in Fruit Wines

Diacetyl is produced as an intermediate during the reductive decarboxylation of pyruvic acid to 2,3-butanediol and is present in fermented liquors such as wine, brandy, and beer [60,61]. The sensory threshold of diacetyl varies depending on the type of wine. High concentrations of diacetyl present (exceeding 5–7 mg/L) are undesirable and contribute to wine spoilage, while diacetyl present in the range of 1–4 mg/L contributes to desirable characteristics such as buttery or butterscotch flavor [61].

**Figure 1:**
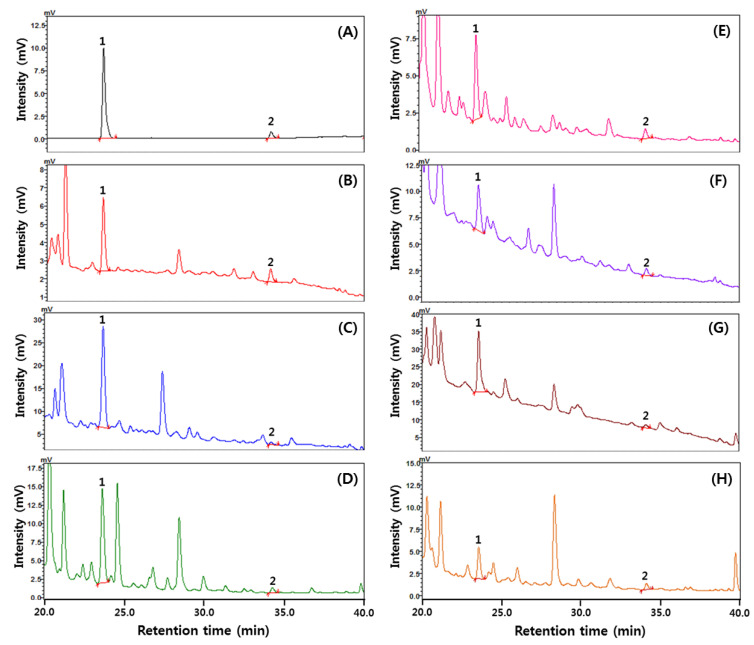
HPLC chromatograms of diacetyl in fruit wines: (**A**) standards of 1 mg/L; (**B**) maesil wine; (**C**) black raspberry wine; (**D**) peach wine; (**E**) apple wine; (**F**) plum wine; (**G**) red grape wine; (**H**) white grape wine. Peak 1, diacetyl; Peak 2, 2,3-hexanedione (IS).

The diacetyl content was the highest in red grape wine, followed by black raspberry, plum, peach, apple, maesil, and white grape wines (Table 4 and Figure 1). The average diacetyl content of fruit wines was 0.432 mg/L, which is higher than for other alcoholic beverages (whiskies, general distilled spirits, beer, and Cheongju, etc.) Diacetyl levels were previously reported to be high, especially in berry wines such as black raspberry wine (2.441 mg/L) and blueberries wine (3.305 mg/L) [62]. Diacetyl content was 9.52 times higher in red grape wine than in white grape wine. It showed a higher trend than for red grape wine (trace–0.616 mg/L) and white grape wine (trace–0.086 mg/L), as reported by Lee et al. [62]. The diacetyl content may vary for the same type of fruit wine, which is thought to be due to differences in the manufacturing method and aging level [60].

In the present study, diacetyl was derivatized into o-phenylenediamine, and quinoxalines generated via the classical Hinsberg reaction were identified and quantified using HPLC-UV [63]. Diacetyl is the most common dicarbonyl compound that binds to SO_2_, especially to HSO_3_^-^, and under the normal pH of wine (pH 2 to 4), it bonds with HSO_3_ to form diacetyl-HSO_3_, whilst this bond is quickly broken at pH 8 or higher [63]. In this study, a derivation is performed after adjusting the pH of wine to 8. Thus, SO_2_ is not expected to affect the diacetyl quantification. The diacetyl content was affected by malolactic bacterial strains, the inoculation rate of malolactic bacteria, contact with active yeast culture, and contact of wine with air during malolactic fermentation. Thus, continuous observation of diacetyl content is considered necessary to maintain fruit wine flavor.

### 3.6. The Content of Ethyl Carbamate in Fruit Wines

Ethyl carbamate is a compound naturally produced during the fermentation and maturation of alcoholic beverages. During the manufacture of wine from stone fruits such as maesil, plum, and peach, cyanogenic glycosides are enzymatically degraded and oxidized to cyanate, which reacts with alcohol produced during fermentation, leading to the formation of ethyl carbamate [15,16]. Urea and citrulline are also precursors participating in the formation of ethyl carbamate. Urea is derived from the catabolism of arginine during ethanol fermentation, and citrulline is generated from the catabolism of arginine by lactic acid bacteria in grape wine and fruit brandies [16]. As ethyl carbamate is classified into group 2A probably carcinogenic to humans’, it is necessary to continuously assess residual levels in alcoholic beverages [5,10,64].

**Figure 2:**
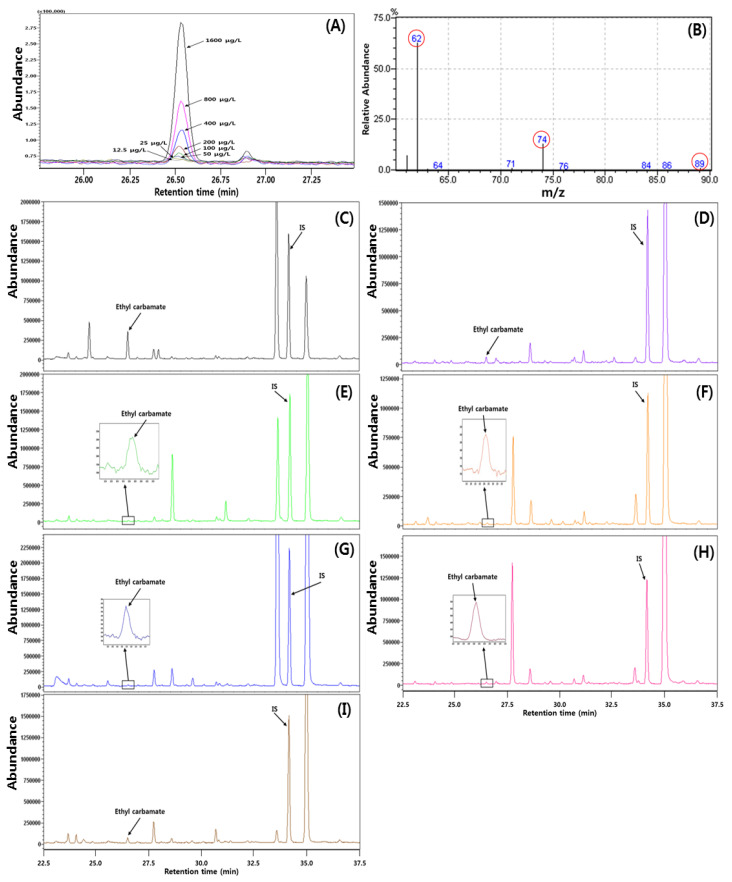
GC-MS-SIM chromatogram (62 m/z) of ethyl carbamate in standards and fruit wines: (**A**) ethyl carbamate of 12.5–1600 μg/L; (**B**) mass spectrum of ethyl carbamate; (**C**) maesil wine; (**D**) black raspberry wine; (**E**) peach wine; (**F**) apple wine; (**G**) plum wine; (**H**) red grape wine; (**I**) white grape wine. IS: internal standard, butyl carbamate.

The content and GC-MS-SIM chromatogram of ethyl carbamate in fruit wines was shown in Table 4 and Figure 2. The ethyl carbamate content of traditional Korean fruit wines ranged from 5.22 to 259.69 μg/kg, and there was a significant difference depending on the type of wine (*p* < 0.05). Maesil wine had the highest ethyl carbamate content (109.37–498.39 μg/kg), 11.02 times (black raspberry wine) to 49.75 times (plum wine) greater than those of other fruit wines (*p* < 0.05). The remaining fruit wines ranked as follows with regard to ethyl carbamate content: black raspberry, apple, white grape, red grape, peach, and plum wines, with no significant difference between these wines (*p* > 0.05). Kim et al. [64] reported that the ethyl carbamate contents in Korean maesil and black raspberry fruit wines were 79.18 μg/L and 1.66 μg/L, respectively, while the ethyl carbamate content in imported grape wine was 2.64 μg/L, which was lower than our results.

In Korea, there are currently no quality control standards for ethyl carbamate as a harmful residual found in fruit wine. When compared to the maximum levels of ethyl carbamate (grape wine: 15–30 μg/L) allowed in Canada, the USA, and the Czech Republic, traditional Korean fruit wines, except for maesil wine, were all within safe levels. In the manufacture of maesil wine, amygdalin contained in maesil is hydrolyzed by β-glucosidase and degraded to HCN [13], whereafter HCN reacts with ethanol generated during fermentation to form ethyl carbamate [65]. The content of ethyl carbamate in wine depends on amygdalin content extracted from maesil fruit and its stone as well as on the fermentation conditions such as temperature, steeping, and ripening time. The ethyl carbamate content was higher in maesil extracts fermented at 25 °C than in those fermented at 15 °C for 1 year [65]. Monitoring of the ethyl carbamate content in maesil liqueur for 1 year showed that the ethyl carbamate content increased to 143–204 μg/kg until 210 days of fermentation time, then decreasing into the end of fermentation [66]. The ethyl carbamate content of maesil wine marketed in Korea was monitored for 1 year and reported as 64–295 μg/L by the Korea Consumer Agency [67]. The measured content was similar to our findings. Apple, peach, and plum seeds also contain amygdalin, and ethyl carbamate is generated when their seeds are macerated or crushed during wine manufacture [68]. The possibility of a large amount of ethyl carbamate remaining following manufacture highlights the need for strict control through government-imposed regulation.

### 3.7. Method Validation

Titration and enzymatic assays were validated and compared as methods for acetaldehyde analysis (Table 5). Accuracy and precision were assessed based on the recovery rate (%) and the RSD (%), respectively. Spiking 10, 50, and 100 mg/L acetaldehyde into maesil, apple, and white grape wines resulted in recovery rates were of 105.33–110.66% via the titration method and 99.26–106.67% via enzymatic assay. An RSD (%) of 0.82–2.99% and 1.08–5.24% was determined via the titration method and enzymatic assay, respectively. Acceptable ranges of recovery for individual analysis are 80–115% and 85–115% at spiking concentrations of 10 mg/L and 100 mg/L, respectively, as per AOAC guidelines [69] and 70–125% as per Korean MFDS guidelines [70]. Acceptable values for RSD (%) are 4% and 5% at spiking concentrations of 10 mg/L and 100 mg/L, respectively in accordance with AOAC guidelines [69], and 20% in accordance with Korean MDFS guidelines [70]. Therefore, both analytical methods were acceptable for acetaldehyde quantification in fruit wines. However, since acetaldehyde quantification in fruit wine with high SO_2_ content is affected by the method applied, the enzymatic assay method is appropriate (Table 3).

Analytical methods for diacetyl and ethyl carbamate quantification were also validated, with specificity, linearity, sensitivity, accuracy, and precision listed in Figure 1 and Figure 2 as well as Table 6. For diacetyl analysis in fruit wines, diacetyl and IS (2,3-hexanedione) in standard solutions and wine samples were separated and identified in HPLC chromatograms (Figure 1), with specificity confirmed via HPLC-UV. The linearity was evaluated through a regression equation of calibration curve (y = 13.686x + 1.3621) and correlation coefficient (R^2^ = 0.9983). Regarding the LOD and LOQ, as expressed, the sensitivities were 0.0007 mg/L and 0.003 mg/L, respectively. The recovery rate at spiking of 1, 5, and 10 mg/L diacetyl standard was 101.15–109.18% in apple wine and 99.24–109.75% in white grape wine, with respective RSDs of 2.02–4.06% and 2.10 to 3.55%, indicating acceptable accuracy and precision in accordance with AOAC guidelines [69] and Korean MDFS guidelines [70].

For ethyl carbamate analysis via GC-MS-SIM, ethyl carbamate and butyl carbamate (as an internal standard) were well separated and identified in standard solutions and wine samples within the range of 12.5 μg/L–1600 μg/L (Figure 2). Ethyl carbamate was confirmed with mass fragments of 62, 74, and 89 m/z in mass spectrum, indicative of higher specificity via GC-MS-SIM. The 62 m/z fragment was more abundant, allowing for higher sensitivity, and was therefore used for ethyl carbamate quantification (Figure 2B). The LOD and LOQ were 0.323 μg/L and 1.067 μg/L, respectively. Linearity was evaluated with calibration curve regression equation (y = 0.0016x + 0.004) and correlation coefficients (R^2^ = 0.9999) in the concentration range of 0.39–1600.00 μg/L. The recovery rate at spiking with 10, 50, and 100 μg/L of standard was 104.49–115.40% in apple wine and 106.33–111.79% in white grape wine, with RSDs of 0.70–1.01% and 1.22–6.13, respectively. The accuracy and precision of ethyl carbamate analysis results were in accordance with these guidelines [69,70]. The obtained method validation parameters indicated that the HPLC-UV and GC-MS-SIM methods were precise and accurate, with high sensitivity in analyzing diacetyl and ethyl carbamate in fruit wines.

Cyanide is phytotoxins as well as one of the precursors of ethyl carbamate, and its content has been regulated in the EU and China. Acetaldehyde and ethyl carbamate are carcinogenic hazards to humans classified by IARC, and some countries, including the EU, Canada, and the U.S.A., regulate the concentrations. However, Korea has not yet set standard regulations for hazardous substances, such as cyanide, acetaldehyde, and ethyl carbamate, in fruit wines. In the present study, trace amounts of hazardous substances were found in traditional Korean fruit wines. Therefore, this study suggested that continuous monitoring and regulations are required for the improvement of quality and safety in traditional Korean fruit wine. Further, it is necessary to assess the hazardous substances in other alcoholic beverages that are frequently consumed in Korea such as *takju, yakju, cheongju*, and *soju*, etc.

## 4. Conclusions

Quality control characteristics (pH, titratable acidity, sulfur dioxide, and diacetyl) and hazardous substance content (cyanide, acetaldehyde, and ethyl carbamate) in five types of traditional Korean fruit wine and two types of imported grape wine were determined in the present study. In addition, method validation was carried out to establish the most appropriate method for the analysis of hazardous substances. The Korean MFDS has set a limit of total sulfur dioxide content in wine, and all fruit wines analyzed in this study met this SO_2_ standard. However, there are no Korean regulatory standards for pH, titratable acidity, diacetyl, acetaldehyde, cyanide, and ethyl carbamate contents. Considering foreign standards, cyanide content in all fruit wines met those of the EU and Chinese. With regard to ethyl carbamate, all wines except for maesil wine met US standard. Taken together, it is necessary to establish a detailed inspection standard and prepare a plan for the quality control of fruit wines in order to reduce the risk of exposure to residual toxic substances. In addition, the method validation parameters for quantitative analyses of acetaldehyde, diacetyl, and ethyl carbamate remaining in fruit wine in this study met the AOAC guidelines, highlighting their utility for the quality and safety control of fruit wines.

## Figures and Tables

**Table 1:** Alcohol content, pH level, and titratable acidity content in fruit wines.

Wine Type		No.	Alcohol Content (%) ^(1)^	pH		Titratable Acidity (g/L) ^(2)^	
				pH	Ave ± SD	Titratable Acidity	Ave ± SD
Traditional Korean fruit wine	Maesil wine	1	12.0	3.14 ± 0.01 ^A (3)^	3.12 ± 0.05 ^cd (4)^	4.68 ± 0.01 ^A^	5.35 ± 0.64 ^ab^
		2	14.0	3.05 ± 0.01 ^B^		5.41 ± 0.08 ^B^	
		3	14.0	3.15 ± 0.00 ^A^		5.95 ± 0.14 ^C^	
	Black raspberry wine	1	12.0	3.05 ± 0.01 ^A^	3.01 ± 0.04 ^d^	7.90 ± 0.22 ^A^	6.68 ± 1.83 ^ab^
		2	15.0	3.03 ± 0.01 ^A^		4.58 ± 0.08 ^B^	
		3	13.0	2.96 ± 0.01 ^B^		7.57 ± 0.20 ^A^	
	Peach wine	1	12.0	3.81 ± 0.01 ^A^	3.76 ± 0.06 ^a^	6.74 ± 0.30 ^C^	7.98 ± 1.18 ^a^
		2	12.0	3.78 ± 0.01 ^B^		9.10 ± 0.04 ^A^	
		3	7.0	3.70 ± 0.01 ^C^		8.09 ± 0.28 ^B^	
	Apple wine	1	16.0	3.84 ± 0.01 ^A^	3.70 ± 0.12 ^ab^	2.01 ± 0.01 ^C^	4.70 ± 2.40 ^b^
		2	12.0	3.61 ± 0.01 ^C^		6.61 ± 0.04 ^A^	
		3	12.0	3.65 ± 0.01 ^B^		5.49 ± 0.06 ^B^	
	Plum wine	1	12.0	3.40 ± 0.01	3.61 ± 0.31 ^ab^	9.08 ± 0.12 *	7.84 ± 1.76 ^a^
		2	12.0	3.83 ± 0.01 *^(5)^		6.60 ± 0.08	
Imported grape wine	Red grape wine	1	14.0	3.50 ± 0.00 ^B^	3.52 ± 0.11 ^b^	6.76 ± 0.18 ^A^	6.12 ± 0.67 ^ab^
		2	13.0	3.42 ± 0.00^C^		6.17 ± 0.14 ^B^	
		3	13.0	3.64 ± 0.01 ^A^		5.42 ± 0.14 ^C^	
	White grape wine	1	14.0	3.25 ± 0.01 ^C^	3.31 ± 0.05 ^c^	6.65 ± 0.21^A^	6.43 ± 0.21 ^ab^
		2	14.0	3.34 ± 0.01 ^A^		6.24 ± 0.15 ^B^	
		3	13.0	3.33 ± 0.01 ^B^		6.42 ± 0.01 ^AB^	

Data are shown as the mean ± SD (*n* = 3). ^(1)^ Alcohol content (%) provided by the wine manufacturer. ^(2)^ Titratable acidity expressed as tartaric acid. ^(3)^ Means with different capital letters ^(A–C)^ in the same column are significantly different within the same type of wine by Duncan’s multiple range test at *p* < 0.05. ^(4)^ Means with different small letters ^(a–d)^ in the same columns are significantly different among the different type of wine by Duncan’s multiple range test at *p* < 0.05. *^(5)^ Indicates a significant difference within the plum wines by Student’s *t*-test at *p* < 0.05.

**Table 2:** Cyanide content in fruit wines.

Wine Type		No.	Cyanide Content (mg/L)	
			Cyanide	Ave ± SD
Traditional Korean fruit wine	Maesil wine	1	0.04 ± 0.01 ^B(1)^	0.06 ± 0.04 ^b(2)^
		2	0.10 ± 0.02 ^A^	
		3	0.04 ± 0.01 ^B^	
	Black raspberry wine	1	0.03 ± 0.02 ^B^	0.08 ± 0.06 ^b^
		2	0.15 ± 0.04 ^A^	
		3	0.06 ± 0.02 ^B^	
	Peach wine	1	0.04 ± 0.02 ^A^	0.06 ± 0.03 ^b^
		2	0.09 ± 0.06 ^A^	
		3	0.07 ± 0.02 ^A^	
	Apple wine	1	0.12 ± 0.02 ^A^	0.13 ± 0.00 ^b^
		2	0.13 ± 0.04 ^A^	
		3	0.13 ± 0.02 ^A^	
	Plum wine	1	0.11 ± 0.05 ^NS(3)^	0.08 ± 0.04 ^b^
		2	0.05 ± 0.01	
Imported grape wine	Red grape wine	1	0.54 ± 0.03 ^A^	0.35 ± 0.029 ^a^
		2	0.50 ± 0.04 ^A^	
		3	0.02 ± 0.02 ^B^	
	White grape wine	1	0.03 ± 0.03 ^A^	0.02 ± 0.00 ^b^
		2	0.03 ± 0.01 ^A^	
		3	0.01 ± 0.02 ^A^	

Data are shown as the mean ± SD (*n* = 3). ^(1)^ Means with different capital letters ^(A,B)^ in the same column are significantly different within the same type of wine by Duncan’s multiple range test at *p* < 0.05. ^(2)^ Means with different small letters ^(a,b)^ in the same columns are significantly different among the different types of wine by Duncan’s multiple range test at *p* < 0.05. ^(3)^ ^NS^ Indicates not significantly different within the plum wines by Student’s *t*-test at *p* < 0.05.

**Table 3:** The contents of sulfur dioxide and acetaldehyde in fruit wines.

Wine Type		No.	Sulfur Dioxide (mg/L)				Acetaldehyde (mg/L)			
			TSO_2_	Ave ± SD	FSO_2_	Ave ± SD	Titration ^(1)^	Ave ± SD	Enzymatic ^(2)^	Ave ± SD
Traditional Korean fruit wine	Maesil wine	1	7.54 ± 0.20	7.54 ± 0.00 ^a(5)^	- ^(3)^	-	24.30 ± 0.48 ^B(4)^	26.01 ± 11.65 ^ab, NS(6)^	23.55 ± 0.70 ^A^	17.40 ± 9.61 ^a6^
		2	-		-		38.42 ± 1.21 ^A^		22.34 ± 0.98 ^A^	
		3	-		-		15.31 ± 0.56 ^C^		6.32 ± 0.16 ^B^	
	Black raspberry wine	1	-	-	-	-	14.26 ± 0.73 ^C^	22.44 ± 10.55 ^ab,NS^	5.03 ± 0.28 ^C^	8.36 ± 4.48 ^a^
		2	-		-		18.72 ± 1.10 ^B^		6.60 ± 0.16 ^B^	
		3	-		-		34.35 ± 1.00 ^A^		13.45 ± 0.32 ^A^	
	Peach wine	1	97.88 ± 0.26 ^B^	148.86 ± 138.08 ^a^	0.17 ± 0.00 ^C^	8.89 ± 13.20 ^a^	42.48 ± 0.73 ^A^	19.80 ± 19.78 ^ab,NS^	71.88 ± 2.49 ^B^	64.91 ± 47.34 ^a^
		2	39.94 ± 0.63 ^C^		2.42 ± 0.10 ^B^		10.81 ± 0.95 ^B^		14.47 ± 1.00 ^C^	
		3	302.75 ± 3.10 ^A^		24.08 ± 0.68 ^A^		6.12 ± 0.45 ^C^		108.38 ± 0.83 ^A^	
	Apple wine	1	-	146.67 ± 84.70 ^a^	-	17.50 ± 0.00 ^a^	16.97 ± 1.66 ^B^	15.06 ± 11.28 ^b,NS^	5.12 ± 0.16 ^C^	54.13 ± 45.34 ^a^
		2	144.95 ± 3.49 ^NS(7)^		-		25.26 ± 0.28 ^A^		94.56 ± 2.05 ^A^	
		3	148.38 ± 1.97		17.50 ± 0.39		2.94 ± 0.28 ^C^		62.71 ± 3.53 ^B^	
	Plum wine	1	14.18 ± 0.70	65.88 ± 73.12 ^a^	0.51 ± 0.00	0.56 ± 0.08 ^a^	19.04 ± 0.55	46.86 ± 39.34 ^a,NS^	5.86 ± 0.00 ^$^	32.76 ± 38.04 ^a^
		2	117.58 ± 1.03 ^$(7)^		0.62 ± 0.10 ^NS^		74.68 ± 1.00 ^$^		59.66 ± 2.52	
Imported grape wine	Red grape wine	1	2.70 ± 0.16 ^B^	7.81 ± 4.44 ^a^	2.42 ± 0.19 ^B^	2.66 ± 0.57 ^a^	22.55 ± 0.73 ^A^	20.80 ± 1.93 ^ab,^*^(6)^	3.92 ± 0.56 ^C^	10.77 ± 7.36 ^a^
		2	10.69 ± 0.62 ^A^		2.25 ± 0.26 ^B^		21.11 ± 0.73 ^B^		18.55 ± 0.98 ^A^	
		3	10.04 ± 0.84 ^A^		3.32 ± 0.26 ^A^		18.72 ± 0.28 ^C^		9.84 ± 0.42 ^B^	
	White grape wine	1	121.33 ± 0.75 ^A^	99.84 ± 20.77 ^a^	14.18 ± 0.61 ^A^	6.19 ± 6.92 ^a^	4.67 ± 0.51 ^C^	8.97 ± 4.16 ^b^	45.12 ± 0.58 ^B^	44.69 ± 7.32 ^a,^*
		2	98.30 ± 0.64 ^B^		2.03 ± 0.45 ^B^		9.26 ± 0.13 ^B^		51.79 ± 1.43 ^A^	
		3	79.88 ± 0.48 ^C^		2.36 ± 0.29 ^B^		12.98 ± 0.83 ^A^		37.16 ± 1.58 ^C^	

Data are shown as the mean ± SD (*n* = 3). ^(1)^ Titration method modified from Korean Food Standards Codex. ^(2)^ Analyzed by enzymatic assay kit. *-* ^(3)^*:* Not detected. ^(4)^ Means with different capital letters ^(A–C)^ in the same column are significantly different within the same type of wine by Duncan’s multiple range test at *p* < 0.05. ^(5)^ Means with different small letters ^(a,b)^ in the same columns are significantly different among the different type of wine by Duncan’s multiple range test at *p* < 0.05. *^(6)^ Indicates a significant difference between titration method and enzymatic assay for acetaldehyde quantification by Student’s *t*-test at *p* < 0.05, and NS; not significantly different at *p* < 0.05. ^(7) $^ Indicates a significant difference of TSO_2_ within the plum wines or the apple wines by Student’s *t*-test at *p* < 0.05, and NS; not significantly different at *p* < 0.05.

**Table 4:** The contents of diacetyl and ethyl carbamate in fruit wines.

Wine Type		No.	Diacetyl (mg/L)		Ethyl Carbamate (μg/kg)	
			Diacetyl	Ave ± SD	Ethyl Carbamate	Ave ± SD
Traditional Korean fruit wine	Maesil wine	1	0.72 ± 0.04 ^A(1)^	0.41 ± 0.27 ^a(2)^	109.37 ± 5.71 ^C^	259.69 ± 209.03 ^a^
		2	0.27 ± 0.00 ^B^		498.39 ± 9.21 ^A^	
		3	0.25 ± 0.00 ^B^		171.31 ± 6.22 ^B^	
	Black raspberry wine	1	5.89 ± 0.27 ^A^	2.67 ± 2.79 ^a^	20.80 ± 3.07 ^B^	23.56 ± 3.64 ^b^
		2	0.87 ± 0.04 ^C^		27.69 ± 2.94 ^A^	
		3	1.25 ± 0.12 ^B^		22.19 ± 0.26 ^B^	
	Peach wine	1	2.68 ± 0.22 ^A^	1.41 ± 1.15 ^a^	7.45 ± 0.36 ^A^	6.06 ± 1.71 ^b^
		2	0.44 ± 0.03 ^C^		4.14 ± 0.65 ^B^	
		3	1.11 ± 0.02 ^B^		6.58 ± 0.54 ^A^	
	Apple wine	1	0.39 ± 0.03 ^C^	0.66 ± 0.34 ^a^	42.66 ± 3.73 ^A^	17.32 ± 21.99 ^b^
		2	1.03 ± 0.08 ^A^		6.06 ± 0.32 ^B^	
		3	0.56 ± 0.01 ^B^		3.24 ± 0.31 ^B^	
	Plum wine	1	2.19 ± 0.13 *^(3)^	1.46 ± 1.03 ^a^	5.82 ± 0.35 *	5.22 ± 0.85 ^b^
		2	0.73 ± 0.08		4.62 ± 0.09	
Imported grape wine	Red grape wine	1	1.89 ± 0.08 ^C^	2.95 ± 1.19 ^a^	8.64 ± 1.51 ^B^	11.35 ± 5.55 ^b^
		2	2.72 ± 0.02 ^B^		17.73 ± 0.88 ^A^	
		3	4.24 ± 0.44 ^A^		7.67 ± 0.69 ^B^	
	White grape wine	1	0.25 ± 0.00 ^B^	0.31 ± 0.15 ^a^	21.34 ± 1.97 ^A^	16.24 ± 5.33 ^b^
		2	0.19 ± 0.02 ^C^		16.69 ± 1.24 ^B^	
		3	0.48 ± 0.03 ^A^		10.70 ± 0.84 ^C^	

Data are shown as the mean ± SD (*n* = 3). ^(1)^ Means with different capital letters ^(A–C)^ in the same column are significantly different within the same type of wine by Duncan’s multiple range test at *p* < 0.05. ^(2)^ Means with different small letters ^(a,b)^ in the same columns are significantly different within the different type of wine by Duncan’s multiple range test at *p* < 0.05. ^(3) *^ Indicates a significant difference within the plum wines are significantly different by Student’s *t*-test at *p* < 0.05.

**Table 5:** Comparison of accuracy and precision of acetaldehyde analysis between titration method and enzymatic assay.

Wine Type		Titration Method		Enzymatic Assay	
	Acetaldehyde	Recovery Rate (%)	RSD (%) ^(1)^	Recovery Rate (%)	RSD (%)
Maesil wine ^(2)^ (No.2)	+10 mg/L	106.87 ± 6.94	1.41	100.92 ± 5.78	1.92
	+50 mg/L	107.35 ± 2.29	1.24	103.52 ± 3.26	2.27
	+100 mg/L	108.01 ± 1.20	0.82	100.93 ± 1.95	1.61
Apple wine (No.1)	+10 mg/L	108.30 ± 4.54	1.26	101.86 ± 4.84	3.49
	+50 mg/L	110.66 ± 2.40	1.49	99.26 ± 3.85	3.61
	+100 mg/L	108.61 ± 3.35	2.50	105.28 ± 5.71	5.24
White grape wine (No.1)	+10 mg/L	105.33 ± 4.54	2.99	100.93 ± 5.78	1.08
	+50 mg/L	107.34 ± 2.40	2.06	102.96 ± 2.25	1.18
	+100 mg/L	108.77 ± 2.05	1.81	106.67 ± 1.63	1.08

^(1)^ Relative standard deviation (RSD) was calculated from triplicate measurements. ^(2)^ Same wine was used for validation of accuracy and precision comparison between two analytical methods.

**Table 6:** Method validation of diacetyl and ethyl carbamate analysis.

		Diacetyl		Ethyl Carbamate		
Instrument		HPLC-UV		GC-MS-SIM		
Sensitivity	LOD ^(1)^	0.0007 mg/L		0.323 μg/L		
	LOQ ^(2)^	0.003 mg/L		1.067 μg/L		
Linearity	Calibration curve	Y = 13.686x +1.3621		Y = 0.0016x +0.004		
	Range of linearity	0.098–19.620 mg/L		0.39–1600.00 μg/L		
	Coefficient regression (R^2^)	0.9983		0.9999		
	Diacetyl			Ethyl carbamate		
		Recovery rate (%)	RSD (%) ^(3)^		Recovery rate (%)	RSD (%)
Accuracy Precision	Apple wine			Apple wine		
	+1 mg/L	105.51 ± 4.21	2.02	+10 μg/L	115.40 ± 10.32	1.01
	+5 mg/L	101.15 ± 4.94	4.06	+50 μg/L	113.03 ± 2.28	0.70
	+10 mg/L	109.18 ± 4.65	3.89	+100 μg/L	104.49 ± 2.13	0.88
	White grape wine			White grape wine		
	+1 mg/L	99.24 ± 2.63	2.10	+10 μg/L	107.83 ± 3.95	4.54
	+5 mg/L	109.75 ± 4.10	3.43	+50 μg/L	111.79 ± 9.50	6.13
	+10 mg/L	108.66 ± 3.95	3.55	+100 μg/L	106.33 ± 5.81	1.22

^(1)^ Limit of detection. ^(2)^ Limit of quantification. ^(3)^ Relative standard deviation (RSD) was calculated from triplicate measurements.

## Data Availability

The data that support the findings of this study are included in the paper.

## Supplementary Materials

The following are available online at www.mdpi.com/article/10.3390/foods11193047/s1, Table S1: Pearson’s correlation matrix between the contents of the acetaldehyde and total sulfur dioxide (TSO2).
