# Supplementary material for: Method Validation and Assessment of Hazardous Substances and Quality Control Characteristics in Traditional Fruit Wines
Source: Foods. 2022 Sep 30;11(19):3047. doi: 10.3390/foods11193047 (PMC9562190; doi:10.3390/foods11193047)
Supplement: Supplementary file 1 [file foods-11-03047-s001.zip › foods-1930640-supplementary.pdf]

**Supplementary Table S1. Pearson's correlation matrix between the contents of the acetaldehyde and total sulfur dioxide ($TSO_2$).**

| | Acetaldehyde (Titration method) | Acetaldehyde (Enzymatic assay) | $TSO_2$ |
|---|---|---|---|
| Acetaldehyde (Titration method) | r = 1 | - | - |
| Acetaldehyde (Enzymatic assay) | r = 0.070 (*p* = 0.593) | r = 1 | - |
| $TSO_2$ | r = -0.127 (*p* = 0.334) | r = 0.931**[1)] (*p* = 0.000) | r = 1 |

[1)] **Correlation by Pearson correlation coefficient is significant at $p<0.01$ (2-tailed).
